# Discovering Aptamers by Cell-SELEX against Human Soluble Growth Factors Ectopically Expressed on Yeast Cell Surface

**DOI:** 10.1371/journal.pone.0093052

**Published:** 2014-03-27

**Authors:** Hsien-Wei Meng, John M. Pagano, Brian S. White, Yoshiko Toyoda, Irene M. Min, Harold G. Craighead, David Shalloway, John T. Lis, Kai Xiao, Moonsoo M. Jin

**Affiliations:** 1 Department of Biomedical Engineering, Cornell University, Ithaca, New York, United States of America; 2 Department of Biomedical Sciences, College of Veterinary Medicine, Cornell University, Ithaca, New York, United States of America; 3 Department of Molecular Biology and Genetics, Cornell University, Ithaca, New York, United States of America; 4 School of Applied and Engineering Physics, Cornell University, Ithaca, New York, United States of America; 5 Department of Radiology, Weill Cornell Medical College, New York, New York, United States of America; 6 Department of Biomedical Engineering, Dongguk University, Seoul, South Korea; University of São Paulo, Brazil

## Abstract

SELEX, the process of selecting aptamers, is often hampered by the difficulty of preparing target molecules in their native forms and by a lack of a simple yet quantitative assay for monitoring enrichment and affinity of reactive aptamers. In this study, we sought to discover DNA aptamers against human serum markers for potential therapeutic and diagnostic applications. To circumvent soluble expression and immobilization for performing SELEX, we ectopically expressed soluble growth factors on the surface of yeast cells to enable cell-SELEX and devised a flow cytometry-based method to quantitatively monitor progressive enrichment of specific aptamers. High-throughput sequencing of selected pools revealed that the emergence of highly enriched sequences concurred with the increase in the percentage of reactive aptamers shown by flow cytometry. Particularly, selected DNA aptamers against VEGF were specific and of high affinity (*K_D_* = ∼ 1 nM) and demonstrated a potent inhibition of capillary tube formation of endothelial cells, comparable to the effect of a clinically approved anti-VEGF antibody drug, bevacizumab. Considering the fact that many mammalian secretory proteins have been functionally expressed in yeast, the strategy of implementing cell-SELEX and quantitative binding assay can be extended to discover aptamers against a broad array of soluble antigens.

## Introduction

Aptamers are specific DNA or RNA oligonucleotides selected from large random sequence pools for their ability to bind specific target molecules. Like antibodies, aptamers can bind with high affinity and specificity to targets ranging from proteins to small molecules [1]–[5]. Aptamers are obtained *in vitro* by a process called SELEX (Systematic Evolution of Ligands by EXponential enrichment)^2^ [1], consisting of repeated rounds of negative selection and enrichment of nucleic acid binders against the molecules immobilized on membranes or beads. Selection of specific aptamers against cell surface proteins has also been performed using intact cells, a method termed cell-SELEX [6]. This method is particularly advantageous if the target molecules are cell surface or transmembrane molecules not amenable to soluble expression and purification, and is necessary if aptamers are selected directly against surface markers specific to certain cell types.

Growth factors, cytokines, and other soluble molecules present in the blood are important signalling molecules, mediating various functions in normal physiology and are also implicated in the onset and progression of many diseases [7]–[10]. Antibodies against serum proteins have been approved or are under investigation as therapeutic agents for various pathological conditions including cancer and inflammatory diseases [11]–[13]. More recently, aptamers as binding reagent have been developed against serum proteins with comparable affinity and specificity as antibodies [14]. To circumvent a difficulty with preparing antigens in solution to be used for selection of binding reagents, we have previously shown that mammalian cell surface molecules ectopically expressed on the surface of yeast cells provided a highly efficient platform for selection of specific, high-affinity antibodies [15], [16]. Rapid discovery of specific antibodies was attributed to the fact that the molecules expressed in yeast cells as a fusion to a cell wall protein, agglutinin, are natively folded, and expressed at high density on cell surface. In addition, the complexity of other naturally expressed cell surface molecules would serve as competitive decoys for rapid depletion of non-specific binders. Here we extend these strategies for antibody selection to the discovery of aptamers against human serum growth factors that are ectopically displayed on the surface of yeast cells using a standard cell-SELEX procedure.

Among the soluble growth factors in serum, we have chosen vascular endothelial growth factor (VEGF), platelet-derived growth factor (PDGF)-A, and interleukin (IL)-6 as target antigens for selection of aptamers. VEGF and PDGF are the essential soluble factors for promoting vasculature growth, and antagonists of the family of growth factors or their receptors have been developed as drugs to treat several types of cancers [17]. Currently available VEGF antagonists exist at least in three different forms: as a monoclonal antibody (*i.e.*, bevacizumab [18]), as an RNA aptamer (*i.e.*, pegaptanib [19]), and as a fusion of VEGF receptor (VEGFR) and the antibody Fc domain (*i.e.*, VEGF-Trap [20]). Currently, pegaptanib is the only FDA-approved aptamer drug and is being used for treating age-related macular degeneration [21]. By ectopically expressing soluble proteins as cell surface molecules in yeast, we were able to use the cell-SELEX strategy to isolate specific DNA aptamers. Using high-throughput sequencing of selected pools generated during SELEX rounds, we isolated DNA aptamers against VEGF having higher binding affinity to VEGF in physiological buffer than previously reported DNA aptamers. By truncating full-length VEGF aptamers into shorter fragments containing all or a part of conserved structural motifs, we identified a minimal key structural motif for these aptamers. Various truncated aptamers against VEGF effectively blocked the capillary tube formation of endothelial cells, an assay used to gauge antagonist potency for blocking angiogenesis, comparable to the effect of the monoclonal antibody, bevacizumab.

The procedure developed in this study can readily be adapted for the discovery of high affinity aptamers against a much larger set of targets by circumventing the challenge of functional expression, purification, and immobilization of mammalian soluble proteins. At the same time, flow cytometry-based assay for quantitative measurement of binding affinity will aid rapid screening of highly enriched sequences revealed by high-throughput sequencing.

## Materials and Methods

### Procedure for yeast cell-SELEX

An initial library of 10^15^ DNA aptamers was screened against yeast cells expressing target proteins. Reactive aptamers against growth factors were dissociated from the cells by incubation with 10 mM dithiothreitol for 10 min at 25°C, which would reduce disulfide bonds between two subunits of agglutinin, thereby releasing Aga2 and the bound aptamers from the cells. The mixture containing the aptamer complex was then cleaned up by a standard phenol-chloroform extraction and ethanol precipitation protocol [22], and amplified by PCR. The primer for the anti-sense strands to aptamers was phosphorylated at the 5′ end and was digested by lambda exonuclease (NEB) for 30 minutes at 37°C. To avoid chemical conjugation of aptamers with fluorescent dyes, we used phycoerythrin (PE)-conjugated streptavidin complexed with biotinylated oligonucleotides (which we call ‘capturing oligonucleotides’) complementary to the constant region of the aptamers. After 4–10 rounds of SELEX, the aptamer pools were subject to high-throughput, next-generation sequencing for bioinformatics analysis (see below).

### Flow cytometry for detecting antigen expression and aptamer binding in yeast

Growth of yeast cells and induction of proteins was identical to the method described previously [15], [16]. To verify the cell surface expression of target proteins, 5 μl of induced yeast culture was harvested, washed, and labeled at 30°C for 30 min with anti-c-Myc antibody 9E10 (ATCC), anti-HA antibody 3F10 (Roche Applied Science), or polyclonal goat anti-VEGF antibody (R&D Systems) as primary antibodies in labeling buffer (PBS, 0.5% bovine serum albumin (BSA), 10 mM MgCl_2_). After washing, cells were subsequently labeled at 4°C for 10 min with goat anti-mouse IgG-PE (Santa Cruz Biotechnology) or rabbit anti-goat IgG-PE (Santa Cruz Biotechnology) secondary antibodies in labeling buffer. Cells were washed and re-suspended in 100 μl of labeling buffer and subjected to flow cytometry (Coulter Epics XL). To measure full-length aptamer binding to yeast, aptamers were first mixed with the biotinylated capturing oligonucleotides complementary to either 5′ or 3′ constant regions, heated at 95°C for 5 minutes, and then gradually cooled down to room temperature for 20 minutes. To measure the binding of truncated aptamers to yeast, truncated variants were synthesized with biotin attached at the 5′ end. Aptamers with capturing oligonucleotides or biotinylated aptamers were then complexed with PE-conjugated streptavidin at a molar ratio of aptamer to streptavidin at 1∶1. After washing, yeast cells were incubated with aptamer/streptavidin complex in aptamer binding buffer (APBB; 10 mM HEPES, pH 7.9, 125 mM NaCl, 25 mM KCl, and 1 mM MgCl_2_). After washing, cells were re-suspended in 100 μl of labeling buffer and subjected to flow cytometry. The Hill equation was fit to data (mean fluorescence intensities vs. concentration of aptamers) using Prism 5 (GraphPad) to determine the equilibrium dissociation constants. The formula for the Hill equation was Y = Y_max_*X/(*K_D_*+X) + NS*X + Y_0_, where X, Y, Y_max_, and Y_0_ correspond to the concentration of aptamers, mean fluorescence intensity from flow cytometry measurements, fluorescence intensity from maximum binding, and background fluorescence with no added aptamers, respectively. The term, NS*X was incorporated to account for linear non-specific binding to yeast cells.

### Flow cytometry for measuring VEGF binding to HeLa cells

HeLa cells were used to measure the inhibition of VEGF binding by selected aptamers. Serially diluted VEGF (recombinant human VEGF165, Syd Labs, Inc., MA) was pre-incubated with aptamers for 30 min at 4°C, and the mixture was then applied to HeLa. VEGF binding to HeLa was detected by polyclonal goat anti-VEGF antibodies (R&D Systems) followed by a rabbit anti-goat IgG-PE-conjugated secondary antibody (Santa Cruz Biotechnology).

### High-throughput DNA sequencing

The aptamer pools after SELEX rounds of 0, 4, 8, 9, and 10 were chosen and subjected to a high-throughput DNA sequencing. A small portion of the PCR products from each selected pool listed above were PCR amplified using the primers that contain a unique 9-mer barcode and the adapters necessary for the HiSeq 2000 (Illumina) sequencing platform. High-throughput sequencing data derived from SELEX pools were filtered, clustered, and analyzed as previously described [23]. Processed sequences were analyzed separately for different pools, and those with >85% sequence identity were placed to the same clusters.

### Gel-shift assay with fluorescein-labeled aptamer

Aptamers were phosphorylated at the 5′ end with adenosine 5′-[γ-thio]triphosphate (Sigma-Aldrich), which were subsequently labeled with 5-Iodoacetamidofluorescein (5-IAF) (Sigma-Aldrich), following a protocol previously described [24], [25]. VEGF and DNA were mixed and incubated in binding buffer (10 mM HEPES, pH 7.9, 125 mM NaCl, 25 mM KCl, and 1 mM MgCl_2_) at room temperature for 2 hours, and were analyzed on a 5% polyacrylamide gel, run at 120 V in 1X TBE at 4°C for 1–1.5 hours. The gel was then imaged by a phosphor-imager (Typhoon 9400, GE Healthcare Life Sciences). The intensity of the bands was quantified by ImageJ (http://rsbweb.nih.gov/ij/, NIH), and the Hill equation was fit to data points to determine the equilibrium dissociation constant.

### Human umbilical vein endothelial cell (HUVEC) capillary tube formation assay

Capillary tube formation assay was performed with a growth factor reduced Matrigel (BD Biosciences). After thawing overnight at 4°C, 50 μL Matrigel was placed into each well of a pre-chilled 96-well plate (Millipore), and the Matrigel was allowed to polymerize for 30 minutes in the 37°C incubator. HUVECs (Lonza, Cat# CC-2517) were placed in the wells coated with Matrigel at 2,000 cells per well in EGM-2 (EGM-2 BulletKit; Lonza) containing 5% FBS. Cells were treated with antibodies (bevacizumab (a kind gift from Dr. Boockvar at Weill Cornell Medical College, New York-Presbyterian Hospital) or isotope control) or aptamers used at 16.6 μM. After 6–12 hours of incubation, three randomly captured microscopic (Observer.Z1, Zeiss) images were recorded. Tube length was quantified by Angiogenesis Analyzer for ImageJ software (http://rsbweb.nih.gov/ij/, NIH). Tube length was assessed by drawing a line along each tube and measuring the length of the line in pixels. The average of three fields was taken as the mean value for each treatment.

## Results

### Cell-SELEX implemented by ectopic expression of human growth factors on yeast cell surface

**Figure 1:**
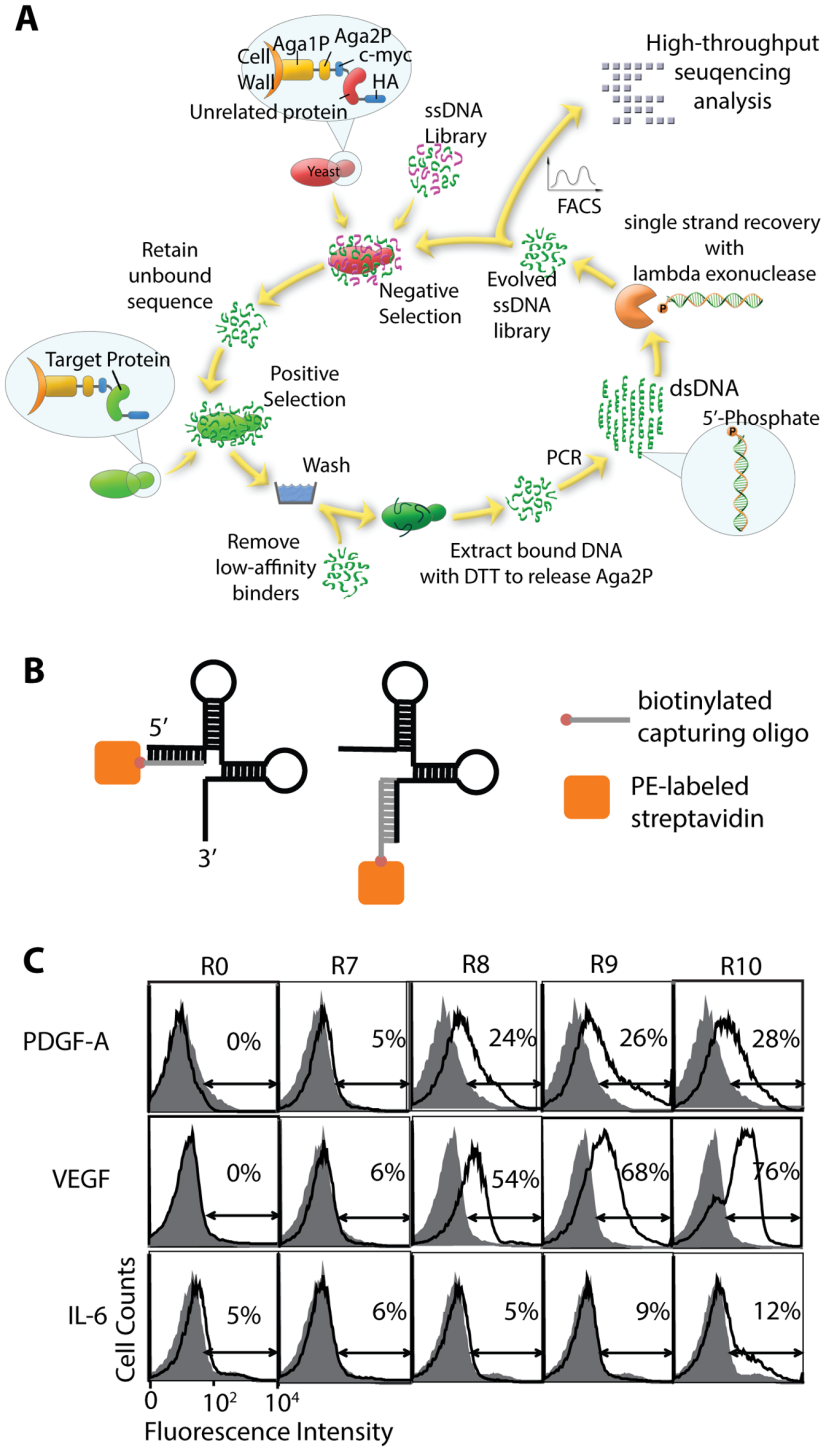
Cell-SELEX procedure implemented by ectopic expression of human soluble growth factors on yeast cell surface. (A) A schematic drawing illustrates the procedure for cell-SELEX. Each round consists of negative and positive selection, PCR amplification, and recovery of aptamer sequences by lambda exonuclease digestion. Human soluble growth factors were displayed on yeast cell surface as a fusion to agglutinin, composed of Aga1p and Aga2p subunits. Surface expression of the protein of interest was confirmed by antibody binding to hemagglutinin (HA) tag (YPYDVPDYA) and c-myc (EQKLISEEDL) tag. Enrichment and affinity of reactive aptamers to target molecules were quantified by flow cytometry. Selected aptamer pools were subjected to high throughput sequencing to isolate highly enriched sequences. (B) Aptamers for flow cytometry experiments were captured by biotinylated oligonucleotides complementary to either 5′ or 3′ constant regions. This DNA complex was mixed with PE-conjugated streptavidin at a 1∶1 molar ratio. (C) Progressive enrichment of reactive aptamers against PDGF-A, VEGF, and IL-6 were confirmed by flow cytometry. Shown are aptamer binding to unrelated proteins (PDGF-B or EGFR) drawn in shaded histograms and to target proteins in open histograms. The percentage of subpopulations defined by arrows is shown for each histogram. Gating was defined throughout this paper to include less than 1% population of unstained cells or cells expressing unrelated targets. Aptamers were used at 200 nM.

DNA aptamers of 100 nucleotides (nt) in length were designed to contain a 60-nt variable region flanked by 20-nt constant regions, which serve as the primer annealing sites for PCR. The growth factors (PDGF-A, PDGF-B, VEGF, and IL-6) were expressed as fusions to the cell wall protein agglutinin for cell surface presentation to enable cell-SELEX procedure (Fig. 1A). A single round of SELEX consisted of negative selection of aptamers with yeast cells expressing unrelated proteins (PDGF-B or epidermal growth factor receptor), positive selection of specific aptamers that bind to target antigens, and PCR amplification. Double-stranded PCR products, composed of a 5′-OH sense strand and a 5′-phosphate anti-sense strand, were treated with lambda exonuclease to digest anti-sense strands and recover single-stranded aptamers [26]. The enrichment of aptamers specific to target antigens was confirmed by an increase in the percentage of binders measured by flow cytometry. In order to avoid direct labeling or chemical modification of aptamers for attaching fluorescent dyes, biotinylated oligonucleotides complementary to either 5′ or 3′ constant regions were used to form a complex with aptamers, which were then added to PE-conjugated streptavidin (Fig. 1B). After eight to ten rounds of SELEX, a substantial (24–76%) sub-population of aptamers exhibited binding to PDGF-A and VEGF antigens (Fig. 1C). Considering the diversity of the initial library (10^15^), the enrichment factor for our cell-SELEX was estimated to be 100-fold per cycle. The percentage of reactive aptamers against IL-6 was lower, reaching ∼12% at the final round.

### Discovery of specific aptamers in accordance with the degree of sequence multiplicity

**Figure 2:**
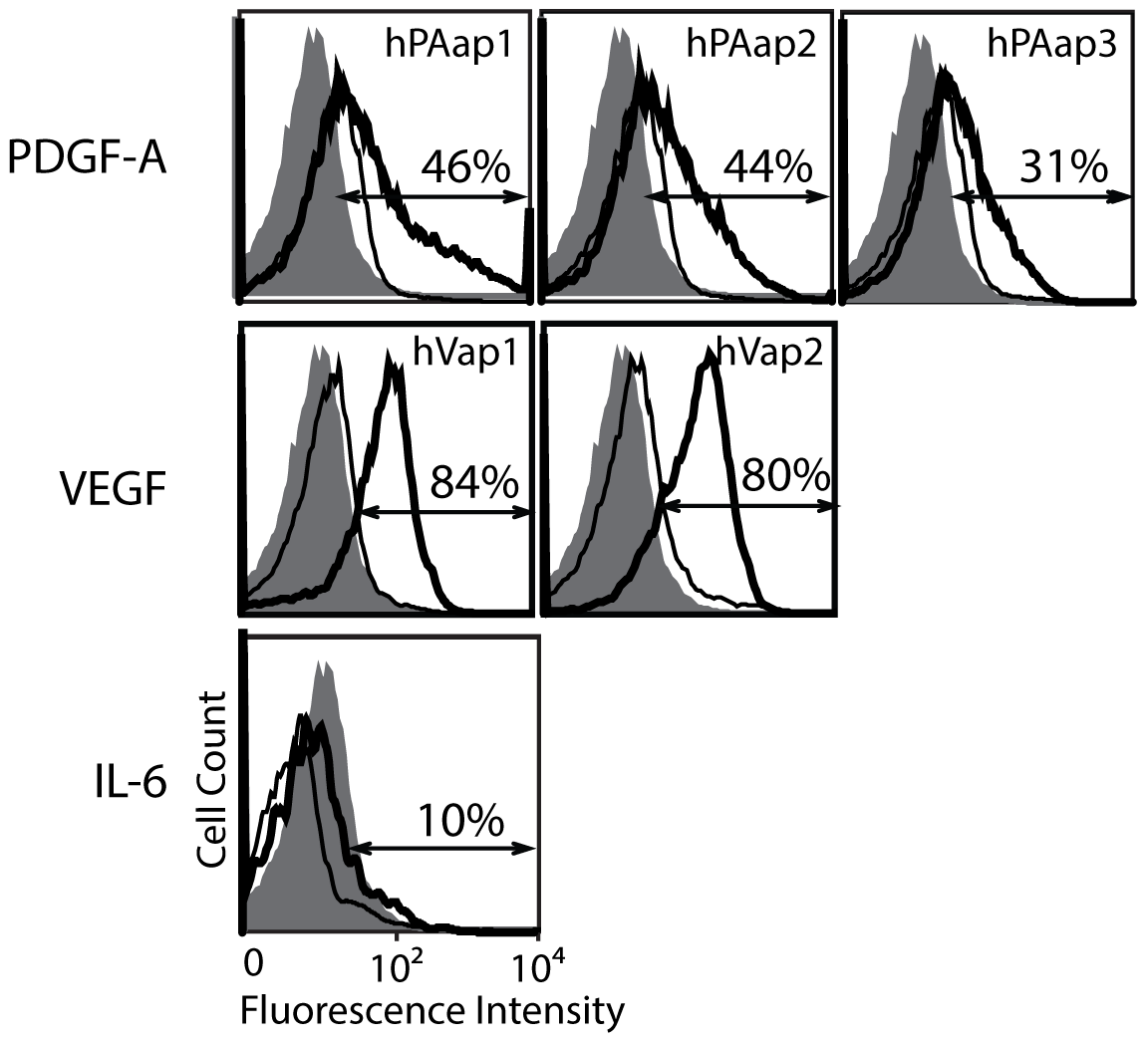
Confirmation of binding specificity of the top-ranked aptamers identified by sequence multiplicity. Highly enriched aptamer sequences were identified by high-throughput sequencing analysis, and tested by flow cytometry for specificity and binding affinity to respective targets. The binding of individual aptamers to respective target is shown as open histograms drawn in thick line and to unrelated protein as open histograms in thin line. Background fluorescence from uninduced yeast cells is shown in closed histograms. The percentage of subpopulations defined by arrows is shown for each histogram.

Using sequence data obtained from the high-throughput sequencing, we calculated the multiplicity (the number of reads) of the sequences (after accounting for sequencing error by clustering) belonging to the different rounds of SELEX (Table 1; Top 300 sequences and multiplicities of aptamers in selected pools are included in Table S1 as an Excel spread sheet.). We expected a correlation between multiplicity and affinity for aptamers, such that the aptamers with tighter binding to targets are more likely to possess higher multiplicity [27]. We chose for high-throughput sequencing analysis the pools prior to SELEX (round 0) and after rounds 4, 8, 9, and 10; the selected pools from the last three rounds were included as there was a substantial increase in reactive binders measured by flow cytometry (Fig. 1C). Highly enriched sequences were also tested individually for binding to respective targets by flow cytometry using biotinylated oligonucleotides complementary to the 5′ end and PE-conjugated streptavidin (Fig. 2). For PDGF-A, the top 10 ranked sequences at round 10 accounted for 63% of the total reads with a gradual decrease in multiplicity from top to bottom. In contrast, the top 2 sequences at rounds 8 to 10 for VEGF (named ‘hVap1′ and ‘hVap2′) accounted for 80% of the reads, with a dramatic decrease in multiplicity for the remaining sequences. Notably, the gradual decrease in the level of binding for the top three PDGF-A aptamers (named ‘hPAap1′ to ‘hPAap3′) (Fig. 2) was correlated with multiplicity, emphasizing quantitative nature of our flow cytometry-based binding assay.

**Table 1:** Sequences and multiplicity of enriched aptamers selected against target molecules.

			Percentage of Total Reads			
Target	Aptamer ID	Sequences of selected aptamers (5'––>3')	R4	R8	R9	R10
PDGF-A	1	CAGAGCAGGGCGCCGTGTCGCATAGTGTTGTCACTCCCCTGGAGGTCCATGCGAAATATG	0.00	13.66	18.52	12.41
	2	GCGGTATTACCGGGGGCCCTCCGGGTACGATTGGGGCTTCCCTATGCGGATCAGTTGGTA	0.00	7.38	11.00	7.40
	3	CGCCTCTTCTCACCGTGTGAGACTCCACGTCTTGATTGCAGGGTGGTTGCTTCCCATGCG	0.00	1.71	1.57	6.85
	4	GTCGCCAAGGGCCGGGCACTGTCTACCGGTATGGTTTAACCTCCCACGCATAGATCGCGT	0.00	3.14	6.36	6.79
	5	GCTAGGGCCGTCAGGCATAGAACCGTCTATTCAACAGAGGATTCTCCCAGATTCGGTGCG	0.00	15.11	10.02	6.64
	6	GAAACGCCGCTGGGCTAACGGGGGGCCCACAGCGTGGGGACGGTGTTTTGATCTATTCTA	0.00	5.49	11.16	5.64
	7	CTCTGGTCGTGAGCTGGGCGGCTATTGCGTGCTAGCCGGTTACTTTTAGTCCCTAATGCG	0.00	2.94	3.50	5.52
	8	GCGTACGTCTCTCTTCAAGACATGCGGACGGGGGCCCGGTACTACGGGGACGATGTTGCG	0.00	3.78	4.88	5.38
	9	GCTGTGGCACACAGGGGGCCCGCAGGTTTTGCAGCGGGGACGATTACTTTTTCGTGCAG	0.00	20.62	12.00	3.47
	10	AAGTTGTCAGGGCAGGTCGGTTGGGCCAACTTGCATGAGTCATGCTTTCCCCGGCGCTCT	0.00	1.36	2.06	3.24
VEGF	1	TAGCCCACTGCATAGAGGTAGCCACGTATGTGGGTGTTACCCATTCGTAGCTACCGGTGA	0.01	79.91	58.21	31.70
	2	AAAGCCCACTGCATAGAGGCCGCTCAACATGCGAGCCGGTGTACTTCAACAGGTATCCT	0.00	2.60	15.81	48.94
	3	GTGGCTCCGCCCACTGCAAAGAGATGGGGGTATCTTCCATCAGTACCATCATCGGTGTAT	0.00	0.17	1.12	4.18
	4	AATCCCTGGGCGTGCAGCCCCTATTGCGTGCTGCCCACTGCAAAGAACGATAGTGGTGTC	0.00	0.32	1.81	4.14
	5	AGGGTCTTCAGAATTGCTTGGGTCCCCACTGGATAGAGCACAAACTGGGCGGTGCATGAC	0.00	12.80	21.07	4.12
	6	CGGAGATAAGTTAGCCCACTGCAGAGAGGCTCTAGTTTGGCATGCCGTCAAGCCGTTGTT	0.00	0.15	0.77	2.26
	7	ATACGTTGGATCCCACTGAATAGAATTTCGCAGCCTGCTCGAACTGGTGTCGCTAATGTT	0.00	0.06	0.23	0.86
	8	CTTAGTCTACCTAAAACAGCCCACTGCATAGAATCTCGGCTTGTACGAGATGGTTGAACA	0.00	0.01	0.02	0.19
	9	TCTTTCGTGTATAATGGCCCACTGCATAGAGTGACTTGTCATGGTGTTTCCATACTTGGT	0.00	0.01	0.01	0.16
	10	TCCCCACTGGTAAGAGCTTGACAAAGCGGTGTTGTTTCATCTCAGTCTTAACTCTGAGTC	0.00	0.01	0.01	0.02
IL-6	1	TCCCACGCATTCTGCACATCGATACTGAGCATCGTACATGATCCCGCAACGGGCAGTATT	0.00		0.03	0.05
	2	TCCCACGCATTCTGCACATCGCGGTTGGGGGGTAAGTGGGGGGTGGTCAGGGGAGGCGG	0.00		0.01	0.02
	3	TCCCACGCATTCTGCACATCCGGTTTTTGCTCCCATAGTGGACCCTATGTCGGCCCATA	0.00		0.01	0.02
	4	TCCCACGCATTCTGCACATCCTGGCTGCCTCCTAACGCGATCTACTGTTCCAATAGCCAC	0.00		0.01	0.01
	5	TAGCCCACTGCATAGAGGTAGCCACGTATGTGGGTGTTACCCATTCGTAGCTACCGGTGA	0.00		0.03	0.01

Underlined sequences correspond to the conserved sequence motif. Sequences were ranked in the order of highest to lowest percentage of total reads of R10 pools, except the top two sequences in VEGF.

### Identification and characterization of aptamers for VEGF

**Figure 3:**
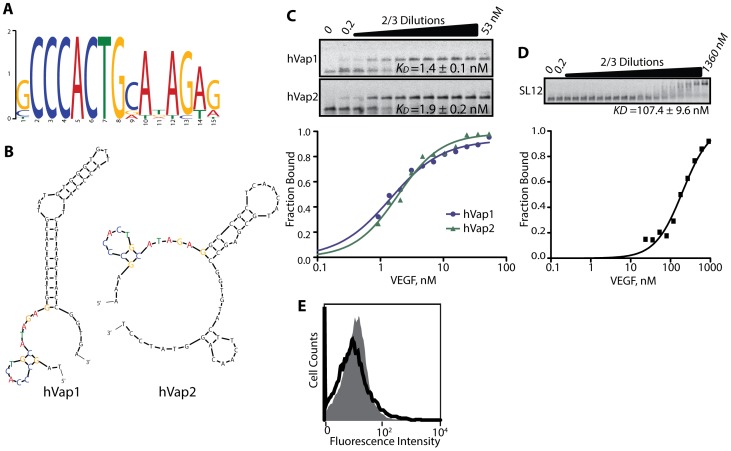
Determining consensus motif, secondary structure, and solution affinity of the aptamers against VEGF. (A) The consensus motif shared among the top ten highly enriched sequences against VEGF was identified using the MEME Suite (http://meme.nbcr.net/meme/). (B) Shown are the secondary structures of the 60-nt variable region of the top two aptamers, hVap1 and hVap2, predicted by Mfold (http://mfold.rna.albany.edu/). (C) Solution affinity of the full-length hVap1 and hVap2 (used at 500 pM) against VEGF was determined by F-EMSA. The fraction-bound values were used to determine the dissociation constant (*K_D_*) of aptamers using the Hill equation. (D) SL12 was labeled at the 5′-end with fluorescein for F-EMSA. (E) Flow cytometry measurement of the binding of the 25-nt aptamer by Potty *et. al.* to yeast cells expressing VEGF (open histogram) and to uninduced yeast cells (filled histogram). The aptamer (used at 200 nM) was biotinylated at the 5′ end to form a complex with PE-conjugated streptavidin.

We then analyzed the highly enriched sequences for the presence of conserved or shared primary (consensus motif) and secondary structures. The top 10 aptamers in round 10 against VEGF were found to contain the consensus motif of [G/C]CCCACTGCA[T/A]AGAG folded into a stem-loop (Fig. 3A&B), although the position of this motif differed within the variable region (Table 1). In contrast, no obvious consensus motifs were found for the PDGF-A aptamers that were present in the majority of the top ranked sequences. As anticipated from the lack of appreciable binding to IL-6 (the binding of the top-ranked aptamer to IL-6 is shown in Fig. 2), none of the sequences selected against IL-6 reached a multiplicity of 0.1% (Table 1). The failure to isolate aptamers against IL-6 is likely due to the lack of high affinity sequences in our starting library, which contained 10^15^ sequences out of a possible diversity of 4^60^ ∼ 10^36^ for the 60-nt variable region, as well as the nature of IL-6 (isoelectric point (pI) 6.2 compared to 9.5 for PDGF-A and 7.6 for VEGF) being less ‘aptogeneic’ to nucleic acid aptamers [28].

**Figure 4:**
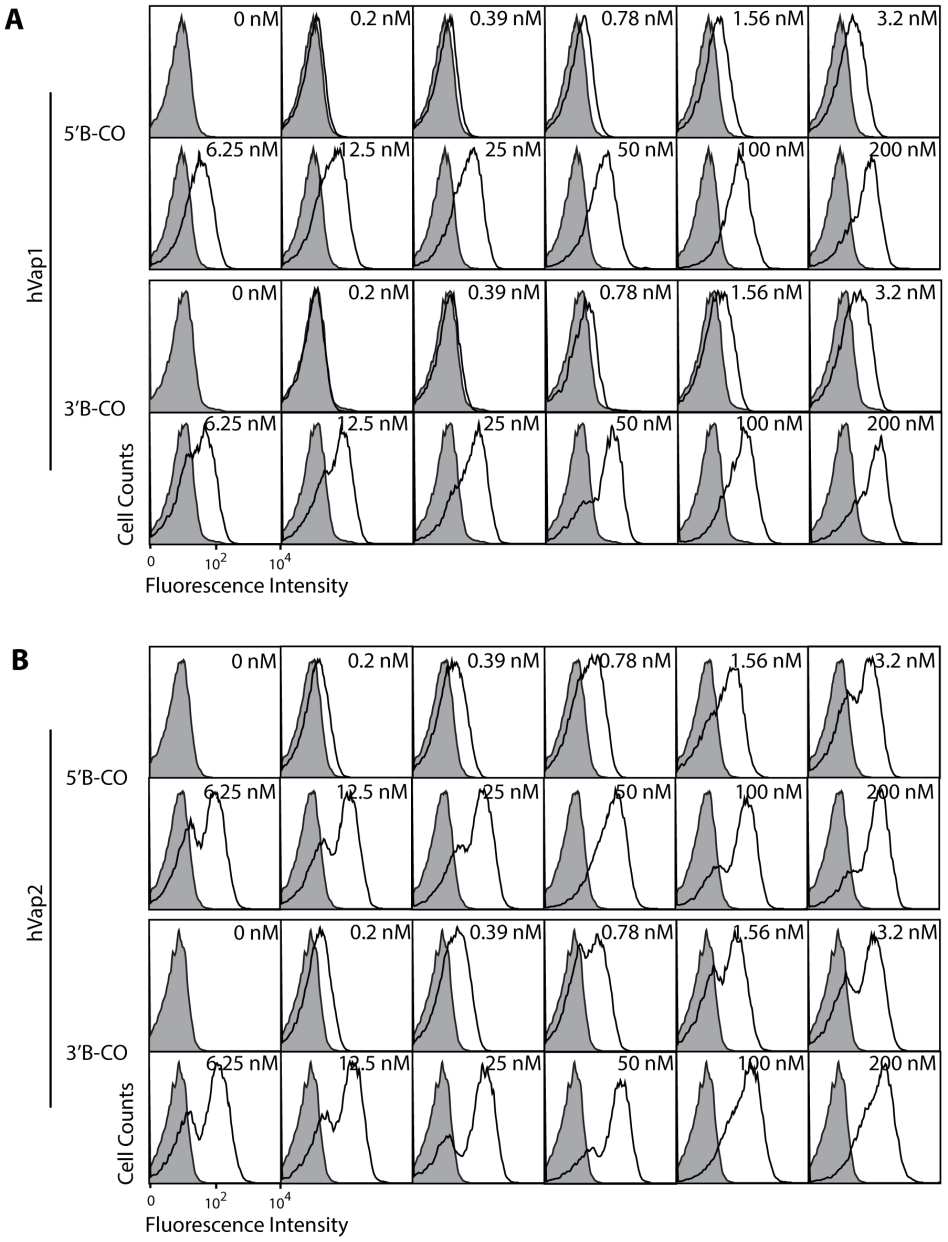
Constant regions are not a part of the active conformation of aptamers. Flow cytometry data are generated with hVap1 (A) and hVap2 (B) captured with biotinylated oligonucleotides complementary to either 5′ (5′B-CO) or 3′ constant regions (3′B-CO). The concentration of aptamers used for labeling yeast is indicated for each histogram. Aptamer binding to yeast cells expressing VEGF is shown as open histogram and to uninduced yeast as filled histogram.

We then chose the top two ranked aptamers against VEGF to further define solution affinity, structural motif, and their potency as VEGF antagonists. To quantitatively measure the solution affinity of aptamers, we performed a fluorescence electrophoretic mobility shift assay (F-EMSA) using fluorescently-labeled VEGF aptamers [24]. The affinity (equilibrium dissociation constant) of hVap1 and hVap2 against VEGF was measured, respectively, to be *K_D_* = 1.4±0.1 nM and 1.9±0.2 nM (Fig. 3C). In comparison, previously reported DNA aptamers against VEGF bound with much weaker affinity by F-EMSA (*K_D_* = 107.4±9.6 nM for SL12 [29]) or did not exhibit measurable binding (the 25-nt DNA aptamer by Potty *et. al.*, [30]) by flow cytometry when tested under equivalent physiological buffer conditions (Fig. 3D&E). The level of binding to VEGF by hVaps captured by the oligonucleotides complementary to either side of the constant region was equivalent (Fig. 4A&B), implying that the 20-nt constant region is not a part of the active structure and that the variable region alone is responsible for binding to VEGF.

**Figure 5:**
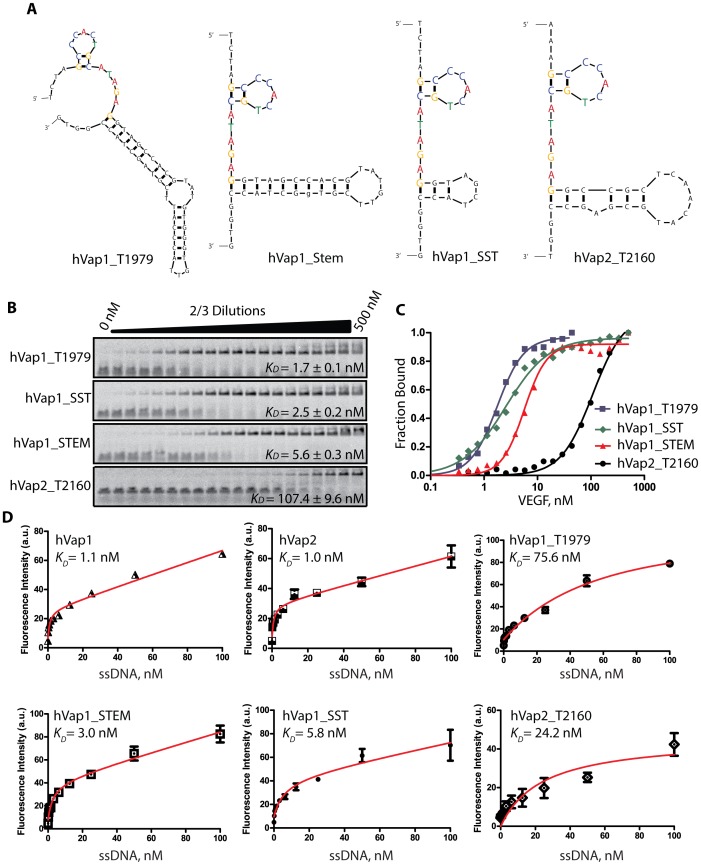
Identification of structural motifs of anti-VEGF aptamers. (A) Full-length anti-VEGF aptamers were shortened to contain the first stem-loop containing the consensus motif shared among all highly sequenced aptamers and a part of the longer stem-loop. (B-C) The binding affinities of all four truncated aptamers were determined by F-EMSA assay (B) and by curve-fitting of the Hill equation to the data (C). VEGF was used at a series of 2/3-fold dilutions starting from 500 nM, and labeled aptamers were at 600 pM. (D) Flow cytometry-based measurement of binding affinity of full-length and truncated aptamers to VEGF. Aptamers labeled with biotin at the 5′ end were used at 0 – 100 nM. Data points are mean ± standard deviation from 2–3 experiments.

Both hVap1 and hVap2 were predicted to have the same stem-loop structure comprised of the consensus motif, followed by a longer stem-loop (Fig. 3B). To further determine if the consensus motif and the predicted secondary structure are the required elements for binding to VEGF, we truncated full-length aptamers into several variants, containing the first stem-loop of the consensus motif and a portion of the adjacent stem-loop (Fig. 5A and Table 2). Again, we performed F-EMSA for each hVap variant and determined the *K_D_* to be 1.7±0.1 nM for hVap1_T1979, 2.5±0.2 nM for hVap1_SST, 5.6±0.3 nM for hVap1_STEM, and 107.4±9.6 nM for hVap2_T2160 (Fig. 5B&C). Equilibrium dissociation constants determined by F-EMSA were in close agreement with those predicted by flow cytometry experiments (Fig.5D), emphasizing quantitative nature of our binding assay.

**Table 2:** Truncated variants of aptamers selected against VEGF.

Aptamer Name	Sequences of truncated aptamers (5'->3')	Length (nt)
hVap1_T1979	TCTAGCCCACTGCATAGAGGTAGCCACGTATGTGGGTGTTACCCATTCGTAGCTACCGGTG	61
hVap1_Stem	TCTAGCCCACTGCATAGAGGTAGCCACGTATGT-------------TC GTGGCTACCGGTG	48
hVap1_SST	TCTAGCCCACTGCATAGAGGTAGC-----------------------------TACCGGTG	32
hVap2_T2160	AAAGCCCACTGCATAGAGGCCGCTCAACATGCGAGCCGGT	40

Dashed lines correspond to the nucleotides that were removed from hVaps. Truncated variants of hVap1 contains 2 nucleotides belonging to the 5′ constant region to provide structural flexibility to the variable region when bound to streptavidin. hVap1_Stem contains the first and second stem-loops without the third stem-loop sequence inserted into the second stem-loop. hVap1_SST contains the first and shortened second stem-loop sequence. hVap2_T2160 contains nucleotides 21–60 of the full-length 100-nt hVap2.

It appears that the first stem-loop in hVap1 is the key structure for binding to VEGF as a significant portion of the second stem-loop could be truncated, applied to hVap1_STEM and hVap1_SST, with less than a 4-fold reduction in binding affinity. It is worth mentioning that the 32-nt hVap1_SST, about half the size of hVap1, bound VEGF with a minimum loss in affinity. Although the first-stem loop is also present in hVap2, which was equally competent as hVap1 in binding VEGF (Fig. 3C), hVap2_T2160 (hVap2 without a possible third stem-loop (Fig. 3B) led to ∼50-fold reduction in affinity to VEGF. This implied that the truncated bases (61–80 nt) of hVap2 are either directly participating in binding or required to adopt the structure needed for binding VEGF.

### Aptamers bind the heparin-binding domain in VEGF

**Figure 6:**
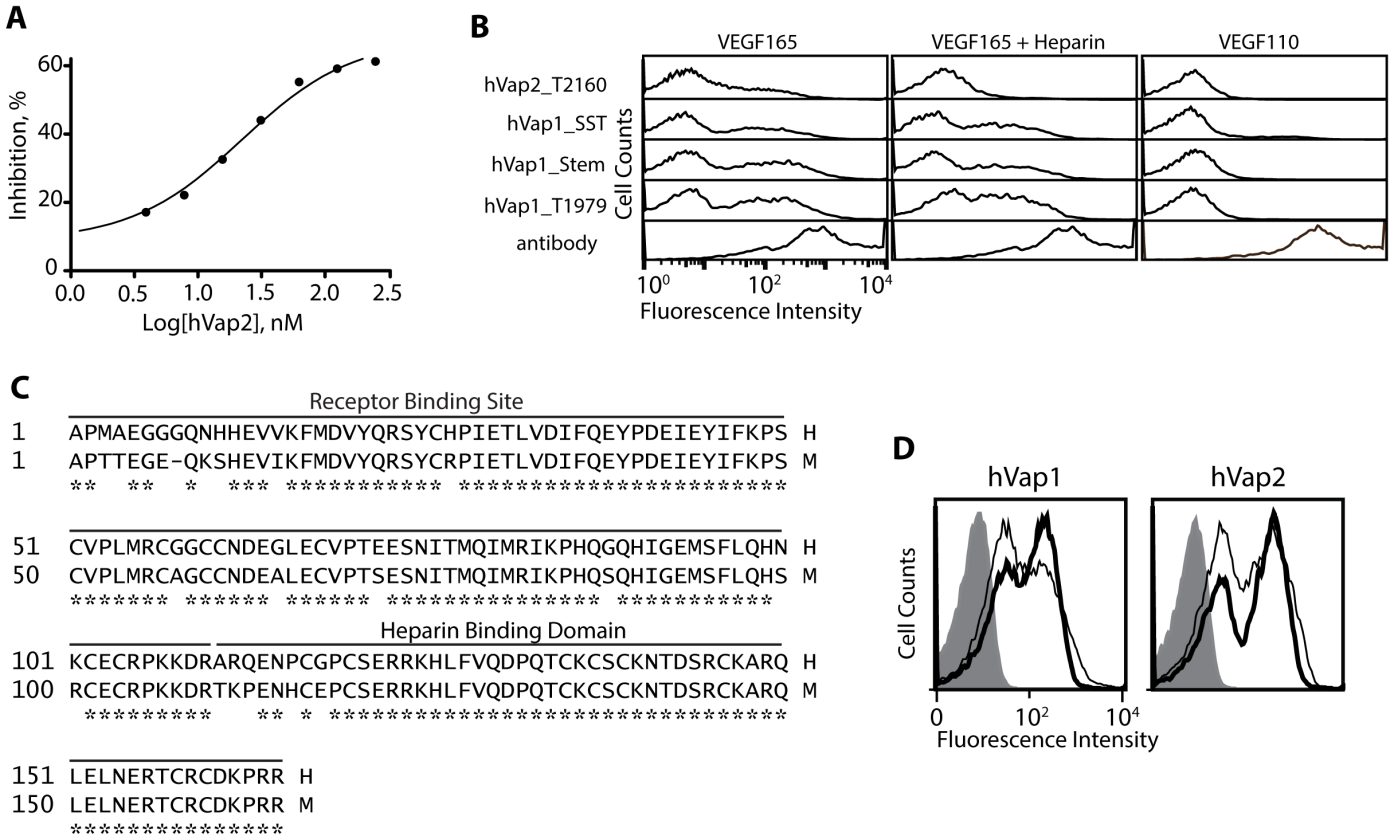
Selected aptamers bind the HBD in VEGF. (A) The potency of anti-VEGF aptamers for the inhibition of VEGF binding was measured as the percent inhibition of VEGF binding to HeLa cells. VEGF at 250 nM was pre-incubated with hVap2 at 0 – 250 nM. VEGF binding to cells was detected by polyclonal anti-VEGF antibody. (B) Anti-VEGF aptamers were tested for binding to yeast cells expressing VEGF165 or VEGF110, which lacks in HBD, with or without post-incubation with 100 nM heparin. Heparin was used to compete with the aptamers for binding to VEGF. VEGF expression was confirmed by polyclonal anti-VEGF antibody. Truncated aptamers were biotinylated at the 5′ end to form a complex with PE-conjugated streptavidin. (C) Sequence alignment of human (H) and murine (M) VEGF. ‘*’ indicates the amino acids conserved between human murine VEGF. Receptor binding domain (residues 1–110 for human VEGF) and heparin binding domain (residues 111–165 for human VEGF) are denoted. (D) Comparison of the level of binding of hVap1 and hVap2 to human (shown as thick open histograms) and murine VEGF (shown as thin open histograms). Aptamer binding to uninduced yeast cells is shown as filled histograms.

VEGF is a major regulator of physiological and pathological angiogenesis [31]. VEGF-A or VEGF itself exists in at least seven homodimeric isoforms, of which VEGF165 (the isoform referred to as VEGF herein) is the dominant species, containing both the receptor-binding domain present in all isoforms of VEGF and a heparin-binding domain (HBD) [31]. HBD has been shown to enhance VEGF binding to VEGFR through its interaction with cell-associated heparin-like glycosaminoglycans and sequestration through extracellular heparin molecules [32], [33]. Pegaptanib is known to recognize the HBD in VEGF, and is likely to inhibit VEGF binding to VEGFR in cells [19]. We examined if our aptamers against VEGF could be used as antagonists to VEGF-mediated signalling. With an increasing concentration of hVap2 pre-incubated with soluble VEGF, we observed a gradual decrease in the level of VEGF binding to HeLa cells known to express receptors for VEGF (Fig. 6A). To further define the structural basis of the inhibition of VEGF binding to VEGFR by our aptamers, we tested if the aptamer binding site is in the receptor binding site or in HBD. When aptamers were tested for binding to VEGF110, devoid of the HBD of VEGF165, all truncated versions of aptamers completely lost the ability to bind VEGF (Fig. 6B), indicating that the epitope for our aptamers is also within the HBD. However, the preformed complex of VEGF with truncated aptamers, except with hVap2_T2160, could not be reduced by the addition of soluble heparin molecules presumably due to tighter binding of VEGF with the aptamers than with heparin. The decrease in hVap2_T2160 binding to VEGF by heparin is ascribed to its lower affinity binding (*K_D_* = 107 nM). HBDs in human and mouse VEGF are highly conserved (50 identical out of 55 amino acids, shown in Fig. 6C), which explains the cross-reaction of hVaps with mouse VEGF (Fig. 6D). Furthermore, we found that hVaps did not show any detectable binding to other heparin-binding proteins (*e.g.*, fibroblast growth factor (FGF)-1 and -2 [34]), providing evidence for the specificity of hVaps toward HBD present in VEGF (Fig. S1).

### hVaps potently inhibit capillary tube formation of endothelial cells

**Figure 7:**
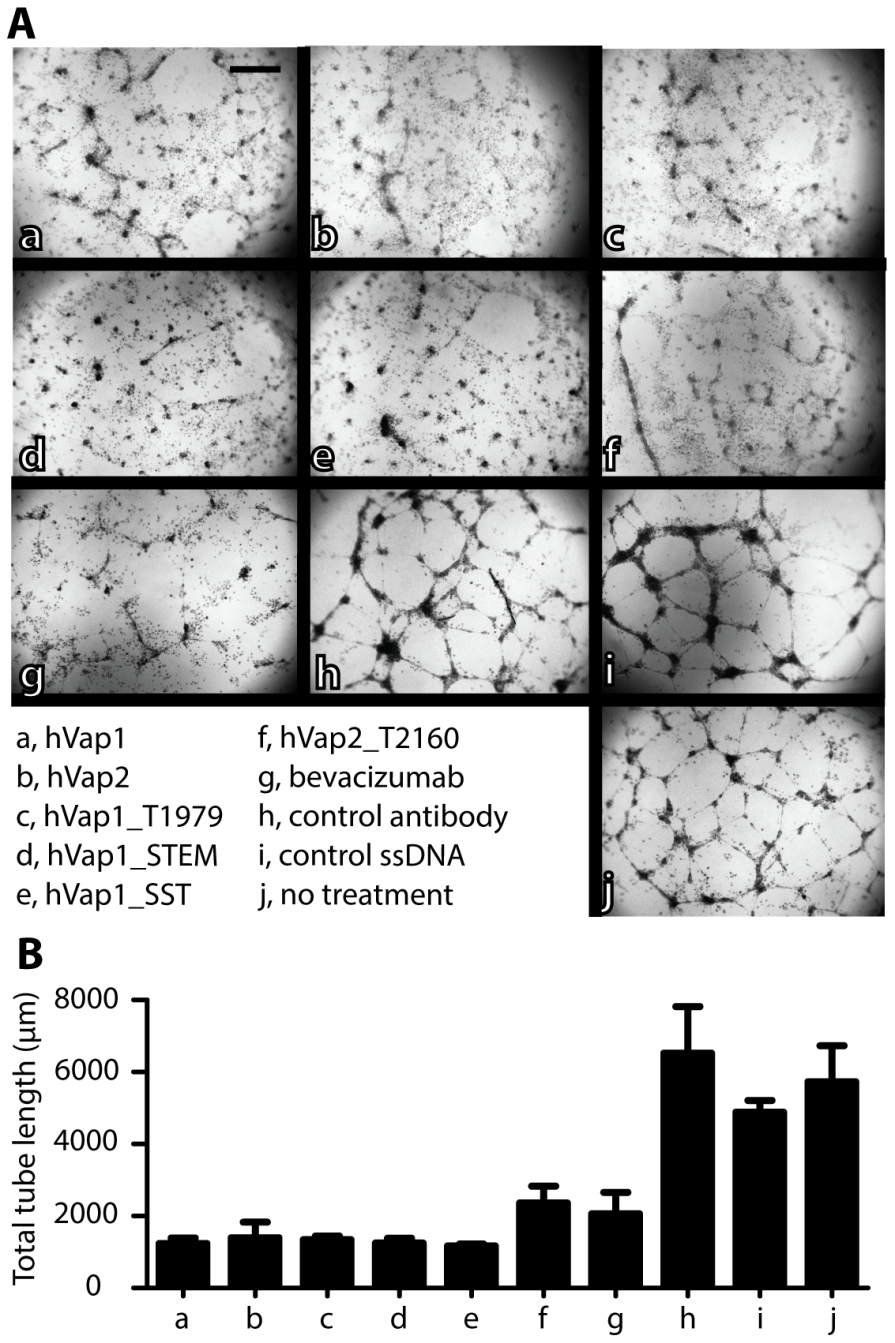
Anti-VEGF aptamers antagonize angiogenesis. Antagonistic potency of anti-VEGF aptamers was measured by a capillary tube formation assay using HUVECs embedded in Matrigel. (A) Shown are the representative images of HUVECs forming capillary tubes after HUVECs were cultured in the presence of anti-VEGF aptamers, bevacizumab, and various controls, all used at same concentrations (16.6 μM). Scale bar  = 100 μm. (B) Total tube length within a randomly chosen microscopic field (25x magnification) was quantified by Angiogenesis Analyzer for ImageJ software. Bar graph shows the mean and standard deviation of the measured total tube length (n = 3).

Next, using an endothelial capillary tube formation assay, we examined the potency of selected VEGF aptamers for their inhibition of VEGF-mediated endothelial vessel formation. Tube formation assay is a well-defined *in vitro* assay that has widely been used to screen for angiogenic and anti-angiogenic factors [35], as it reciprocates the process of physiological angiogenesis. HUVECs plated at sub-confluent densities developed complex mesh-like structure patterns when cultured without antagonists, control antibodies, or control ssDNA (Fig. 7). When treated with bevacizumab (16.6 μM), the HUVECs formed discontinuous but visible sprouting capillary tube patterns. Remarkably, the inhibitory effect of hVaps (16.6 μM) was more potent with little capillary sprouting patterns, resulting in a reduction of tube length by 60–80% over controls (Fig. 7B). Therefore, we conclude that our newly selected VEGF aptamers may possess a therapeutic potential for diseases that can be treated or alleviated by anti-angiogenesis therapy.

## Discussion

Serum growth factors are important therapeutic and diagnostic targets, and peptides, antibodies, and more recently aptamers have been developed that can detect and antagonize the roles mediated by these growth factors. However, it is challenging to functionally express and immobilize these soluble molecules and to maintain their native conformation through repeated rounds of *in vitro* selection. Furthermore, conventional methods for determining binding affinity and specificity of candidate aptamers are tedious and incompatible with rapid screening of highly enriched sequences identified by high-throughout sequencing analysis. To address these limitations, we developed a new cell-SELEX approach that circumvents the need for soluble expression, purification, and immobilization of target molecules, and is well-suited for quantitative and rapid screening of candidate aptamers. This is implemented by ectopic expression of soluble growth factors on the cell surface of yeast, and flow cytometry-based assay to quantify binding affinity and specificity of reactive aptamers. The aptamers selected against VEGF using this approach were found to possess high affinity and selectivity, and blocked endothelial tube formation as potently as the monoclonal antibody drug, bevacizumab. Although a number of DNA aptamers against VEGF have been reported, the fact that the aptamers selected in this study are the only high affinity DNA binders in physiological buffer conditions underscore the efficiency of our SELEX strategy.

Compared to the proteins immobilized to planar membranes or polymer beads, the proteins expressed on the yeast cell surface provide an optimal condition for rapidly depleting non-specific binders and enriching for high affinity binders. This is due to the fact that the library undergoes negative selection with all natively expressed yeast proteins and the positive selection is specific to target protein. Proteins displayed on the surface of yeast cells via a fusion to agglutinin are natively folded, as required to be released through a secretory pathways rather than being degraded intracellularly [36]. Furthermore, surface expression of target proteins in living cells should prevent the targets to be non-uniformly displayed to the extent that can lead to local clustering or aggregation. This has been recognized as a potential problem in SELEX as targets immobilized onto planar membranes or beads are prone to selecting non-specific aptamers due to their binding to multiple targets in a cooperative manner [37]. Finally, the net negative charge of the yeast cell wall, composed primarily of polysaccharides, creates a stringent interaction environment for negatively charged nucleic acid aptamers and proteins present on cell surfaces. These conditions are likely responsible for the successful discovery of aptamers against VEGF and PDGF-A as specific and high affinity binders.

High-throughput sequencing analysis of different aptamer pools revealed a quantitative correlation between aptamer multiplicity and binding affinity. Candidate aptamers against VEGF are dominated by the top two ranked aptamers, hVap1 and hVap2, accounting for 80% of the total reads starting from the 8th round SELEX pool. These two aptamers were confirmed to be high affinity binders to VEGF and potent antagonists to VEGF-mediated angiogenesis. Although the 7th round pool was not subjected to high-throughput sequencing, a dramatic increase in the percentage of reactive aptamers from the 7th to the 8th round pool implied that at least 8 rounds of SELEX were necessary for the discovery of the high affinity aptamers. This corresponds to an enrichment factor of 100-fold per cycle, because the diversity of the starting library is 10^15^. It is also interesting to note that while hVap1 was dominant from the 8th round, hVap2 progressively enriched from the 8th to the 10th round of SELEX. In contrast, aptamers against PDGF-A were found to be weaker binders (with *K_D_* ∼ 4 μM; Fig. S2) consistent with the lack of dominant species revealed by sequence multiplicity. The power of large-scale sequencing for aptamer discovery is also apparent in aptamer pools selected against IL-6. Although flow cytometry revealed gradual enrichment of a reactive population at the 10th round, no single aptamer accounted for more than 1% of the total reads.

The top ranked aptamers by sequence multiplicity accounting for more than 95% reads were found to share the same consensus motif of 15 bases, which adopted a short hairpin structure. Variants of the full-length VEGF aptamers, containing the consensus motif and a truncated form of the adjacent stem-loop, were found to retain almost full capacity binding to VEGF. We speculate that the stem-loop adjacent to the consensus motif may affect binding to VEGF either by direct contact with VEGF or influencing the structure of the consensus motif. The shortest version of the 32-nt VEGF aptamer, consisting of the consensus motif and an adjacent short stem-loop structure, retained affinity to VEGF with less than 2-fold reduction from the full-length 60 nt aptamers. Furthermore, in an endothelial capillary tube formation assay these shortened versions were as potent as the full-length aptamers, which themselves were equally or more effective than the anti-VEGF monoclonal antibody, bevacizumab. Therefore, the VEGF aptamers developed in this study may find a therapeutic use in diseases whose progression is critically dependent on VEGF-mediated vasculature growth.

Affinity measurement by F-EMSA and flow cytometry demonstrated that the aptamers discovered in this study had higher affinity (*K_D_* < 10 nM) relative to two previously isolated DNA aptamers: 1) SL12, a truncated version of the original 66-nt aptamer that was measured to have *K_D_* = 130 nM [38] and 2) the 25-nt aptamer by Potty *et. al.*, which bound VEGF at a lower affinity with increasing salt concentrations, approaching a μM *K_D_* at physiological conditions. Unlike the reported *K_D_* value of 5 nM for SL12, which was determined by surface plasmon resonance [29], the affinity estimated by our F-EMSA assay was ∼100 nM, closer to the affinity of the full-length 66-nt aptamer [38]. The binding of the 25-nt aptamer by Potty *et. al.* could not be detected by flow cytometry performed under physiological buffer conditions, likely because the detection limit of our assay is *K_D_* ∼ 1 μM. Although all these DNA aptamers and the RNA aptamer (pegaptanib) bound VEGF with a wide-range of affinity (sub-nanomolar to micromolar *K_D_*), they all target the HBD, which has a pI of 9 and tends to be a dominant site for interaction with aptamers. One may need to use VEGF devoid of the HBD, such as VEGF110, to isolate aptamers that bind the receptor binding domain to isolate aptamers that can antagonize all isoforms of VEGF.

Overall, the strategy developed in this study, enabling cell-SELEX for selecting aptamers against soluble proteins, rapid enrichment of high affinity and specific binders, and quantitative binding assay can be readily extended to the discovery of aptamers against a larger set of antigens. The fact that quantitative estimation of the binding affinity between aptamers and target proteins can be achieved by flow cytometry has a significant advantage over conventional methods such as EMSA, which require purified proteins and labeled nucleic acids. Flow cytometry also allows a set of yeast cells expressing hundreds of different target proteins to be tested for binding to multiple candidates with high multiplicity, thus ensuring discovered aptamers to possess high affinity and specificity to targets.

## Supporting Information

Figure S1 **hVaps are specific to heparin-binding domain (HBD) present in VEGF.** Histograms show the binding of hVap1 (A) and hVap2 (B) to VEGF, FGF1, FGF2, and uninduced yeast cells (labeled as ‘SD’).
(JPG)

Figure S2 **Affinity measurement of PDGF-A aptamer by flow cytometry.** (A) Fluorescently-labeled aptamer (hPAap1) was used to measure binding to PDGF-A expressed in yeast. (B) Fluorescence intensity values corresponding to the binding of hPAap1 at 0 – 10 μM were used to estimate equilibrium dissociation constant (*K_D_*) by curve-fit with the Hill equation.
(JPG)

Table S1 **Top 300 sequences and multiplicities of aptamers in selected pools.**
(XLSX)
