# Supplementary figures and images for: Discovering Aptamers by Cell-SELEX against Human Soluble Growth Factors Ectopically Expressed on Yeast Cell Surface
Source: PLoS One. 2014 Mar 27;9(3):e93052. doi: 10.1371/journal.pone.0093052 (PMC3968096; doi:10.1371/journal.pone.0093052)

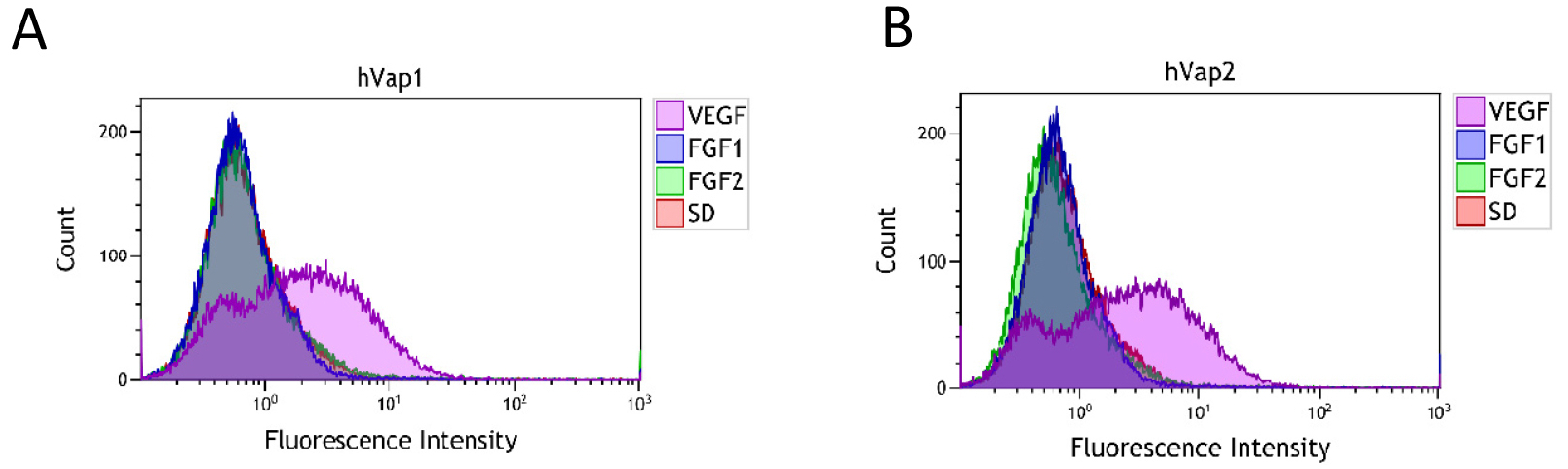

Supplement: Figure S1 — hVaps are specific to heparin-binding domain (HBD) present in VEGF. Histograms show the binding of hVap1 (A) and hVap2 (B) to VEGF, FGF1, FGF2, and uninduced yeast cells (labeled as ‘SD’). (JPG) [file pone.0093052.s001.jpg]

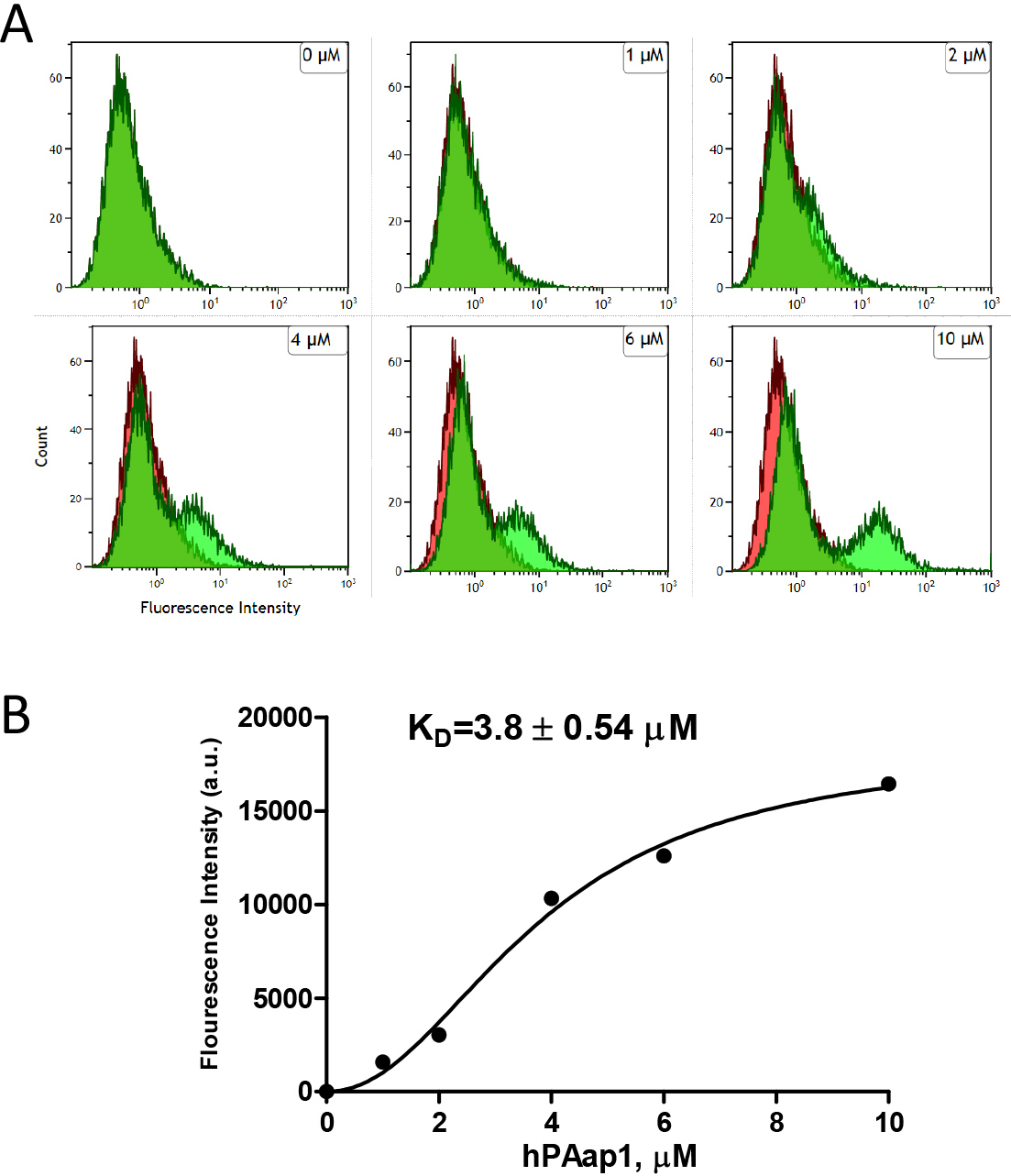

Supplement: Figure S2 — Affinity measurement of PDGF-A aptamer by flow cytometry. (A) Fluorescently-labeled aptamer (hPAap1) was used to measure binding to PDGF-A expressed in yeast. (B) Fluorescence intensity values corresponding to the binding of hPAap1 at 0 – 10 μM were used to estimate equilibrium dissociation constant (KD) by curve-fit with the Hill equation. (JPG) [file pone.0093052.s002.jpg]
